# Accelerated Fabrication of Fiber-Welded Mesoporous Cotton Composites

**DOI:** 10.1021/acsomega.3c09797

**Published:** 2024-02-09

**Authors:** Peyton J. Johnson, Anders J. Gulbrandson, Nathaniel E. Larm, Christopher D. Stachurski, David P. Durkin, Paul C. Trulove

**Affiliations:** † Department of Chemistry, United States Naval Academy, Annapolis, Maryland 21402, United States

## Abstract

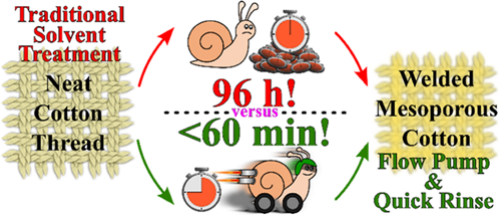

Natural fiber-welded (NFW) biopolymer composites are rapidly garnering industrial and commercial attention in the textile sector, and a recent disclosure demonstrating the production of mesoporous NFW materials suggests a bright future as sorbents, filters, and nanoparticle scaffolds. A significant roadblock in the mass production of mesoporous NFW composites for research and development is their lengthy preparation time: 24 h of water rinses to remove the ionic liquid (IL) serving as a welding medium and then 72 h of solvent exchanges (polar to nonpolar), followed by oven drying to attain a mesoporous composite. In this work, the rinsing procedure is systematically truncated using the solution conductivity as a yardstick to monitor IL removal. The traditional water immersion rinses are replaced by a flow-through system (i.e., infinite dilution) using a peristaltic pump, reducing the required water rinse time for the maximum removal of IL to 30 min. This procedure also allows for easy in-line monitoring of solution conductivity and reclamation of an expensive welding solvent. Further, the organic solvent exchange is minimized to 10 min per solvent (from 24 h), resulting in a total combined rinse time of 1 h. This process acceleration reduces the overall solvent exposure time from 96 to 1 h, an almost 99% temporal improvement.

## 1 Introduction

Natural fiber welding (NFW) of cellulosic biopolymer is a value-added process for producing all-cellulose composites with attractive properties, such as remarkable strength, resiliency, and recyclability.^1,2^ In short, the technique utilizes warm ionic liquid (IL), typically 1-ethyl-3-methylimidazolium acetate (EMImAc), to partially dissolve the outermost layers of biopolymer,^3−7^ followed by rinsing to remove the IL and reconstitute a new material.^8−11^ During reconstitution, adjacent discrete threads are welded together as the hydrogen bonds within the biopolymer reform. Further studies revealed the ability to employ predissolved biopolymer in the IL welding solution, resulting in greatly enhanced welding speeds and a distributed extraneous cellulose layer across the composite after rinsing and drying the welded material.^12^ This process is exceptional for recycling waste biopolymers and adding value to commercial materials. Indeed, commercialization of NFW is well underway,^13^ and the consumer market may be flush with recycled, fiber welded biopolymer materials in the coming years. For this reason, the process for industrializing fiber welded materials should be optimized to maximize solvent and temporal efficiency and minimize the waste of expensive welding solutions (see Green Chemistry principles 1 and 6 regarding waste prevention and energy efficiency, respectively^14,15^).

Recently, a modified NFW technique was demonstrated for the creation of mesoporous and nonderivatized all-cellulose aerogels and xerogels.^16,17^ This modification requires a facile refactoring of the solvent washes post weld; a polar solvent, primarily H_2_O, is first used to remove the welding solution, followed by sequential solvent exchanges to a nonpolar solvent, like cyclohexane (CH), prior to drying.^18,19^ It is postulated that drying welded materials from a polar (and hydrogen bonding) solvent is disruptive, causing induced pores to collapse, whereas drying from a nonpolar solvent is less disruptive and retains the composite mesoporosity and surface area. This hypothesis agrees with the current understanding regarding water removal from, and subsequent collapse of, porous cellulosic materials produced from alternate methods.^20−22^ Unfortunately, the state-of-the-art procedure for making mesoporous NFW composites is excessively time- and solvent-intensive (requiring over 1 L of distilled water and hundreds of mL of organic solvents for ca. 1 g of NFW material). Excluding the weld time, traditional IL extraction with H_2_O requires 24 h (i.e., replenishing the H_2_O after residence times of 10, 20, 30, 90, 180, and 1110 min), followed by sequential solvent exchanges every 24 h (typically 3 solvents after H_2_O: isopropyl alcohol (IPA), 2-butanone (2B), and CH) for a total post-weld solvent residence time of 96 h. These long processing times are detrimental to both research and industrial efforts, particularly when preparing multiple identical products for repeat measurements or small variable adjustments.

Herein, we refactor the solvent exchange portion of the fiber welding methodology toward process acceleration, aiming to minimize solvent and temporal load.^23,24^ The welding conditions used herein are similar to those of prior studies (pure EMImAc as the welding solution and 60 min of welding time at 60 °C) to allow comparisons between our composites and those in the published literature. We optimize the initial H_2_O rinse time by performing the traditional procedure for preparing mesoporous cellulose aerogels while measuring solution conductivity after each H_2_O rinse during the first 24 h. This preliminary assessment yields baseline information regarding the rate of IL removal and is crucial for verifying the absence of residual IL in the welded composite. We then tune two parameters, the H_2_O replenishment frequency and submersion time, while measuring solution conductivity to optimize H_2_O rinse conditions. Next, we perform truncated post-H_2_O solvent exchanges with 5, 10, 15, and 30 min durations and compare the resulting Brunauer–Emmett–Teller (BET) surface areas and pore size distributions to those of a composite prepared by using the traditional 24 h rinses. Finally, we combine a reciprocating pump-based H_2_O flow rinse (and in-line conductivity measurements to confirm adequate IL removal) with the truncated solvent exchanges to bring the total rinsing time from the traditional 96 h to a far more reasonable time of 1 h. The time savings are of obvious benefit in a laboratory setting, and the simple and inexpensive flow-through pump system can be directly converted to industrial situations.

## 2 Experimental Section

### 2.1 Materials

Commercially available cotton Aida cloth (Sensations 22 Count Aida cloth cross stitch fabric), cotton yarn (Coats and Clark mercerized cotton thread, CA0011, S975, 1004), 1-ethyl-3-methylimidazolium acetate (EMImAc, Io-Li-Tec, IL-0189-TG, 95%, nominal water content by KF titration is 0.28 wt %), isopropanol (IPA, Aldrich, 190764 ≥99.5%), 2-butanone (2B, Aldrich, 360473, ≥99.0%), methanol (MeOH, Aldrich, 34860, ≥99.9%), and cyclohexane (CH, Aldrich, 227048, 99.5%) were used as received. Used organic solvents were recycled via distillation over magnesium sulfate. Ultrapure 18.2 MΩ·cm water was obtained from a Milli-Q filtration system.

### 2.2 Instrumentation

A Cole Parmer Masterflex L/S pump (model 7554–90) with a poly(tetrafluoroethylene) (PTFE) tubing pump head (77390–00) was used for the flow-through system. Masterflex 4 mm PTFE tubing (38 cm) and other PTFE tubing were connected using a Masterflex fitting (dimensions 1/8″ ID × 3/32′′ ID, MFLX40614–55) for use in the flow-through system (Figure S1). A 47 mm continuous flow filtration kit (Southern Labware, 167210–36) was used to hold a welded composite in place for the flow-through system. Modifications of the kit include the tubing listed above and a steel filter (1/4 mm width).

### 2.3 Characterization

A YSI 3200 conductivity meter was used to measure the conductivity of the H_2_O rinse solutions. Post weld, immersion solutions were mixed on a Barnstead Lab-Line multipurpose rotator table at speed setting 6 (ca. 30 rotations per min). An N_2_-filled glovebox was used during the fiber welding process to maintain a dry environment (e.g., <1 ppm atmospheric H_2_O content). The Brunauer–Emmett–Teller (BET) surface area, isotherm hysteresis, and pore size/distribution were analyzed by gas physisorption using a Micromeritics ASAP 2020. Samples were sputtered with a thin (i.e., sub-10 nm) layer of gold and then imaged using a TESCAN MIRA3 FEI scanning electron microscope (SEM) operated at an accelerating voltage of 10 kV.

### 2.4 Aida Cloth Preparation

For conductivity measurements, sheets of Aida cloth were cut into 3 in. × 4 in. pieces (ca. 1.25 g) and sewn along the short edges using cotton thread to create cylinders that fit easily within a 240 mL Qorpak bottle (Figure S2). For flow-through system treatment, a 1 in. × 1 in. piece of Aida cloth (ca. 0.10 g) was used so that it would fit within the rinsing apparatus. In both cases, the cloth was dried in vacuo at 60 °C for at least 24 h to remove residual water prior to use.

### 2.5 Fiber Welding Procedure

All welding solutions were maintained within an N_2_-filled glovebox to prevent water exposure. A 240 mL Qorpak bottle was filled with about 200 mL of EMImAc and heated to 60 °C inside a custom aluminum furnace (Figure S3). Dry Aida cloth pieces or assemblies were submerged in the EMImAc for 60 min of quiescent welding. Subsequently, the cloth was retrieved and placed in a clean and dry 240 mL Qorpak bottle for removal from the glovebox.

### 2.6 Traditional Rinsing Procedure

The traditional rinse for converting welded cotton cloth into mesoporous cotton was derived from a previous report.^17^ In short, the EMImAc-treated cloth was submerged in ca. 200 mL of H_2_O for iterative rinses lasting 10, 20, 30, 90, 180, and 1110 min. Following this, the welded cloth was transferred to a 200 mL Qorpak with 100 mL of IPA for 24 h, then 100 mL of 2B for 24 h, and finally 100 mL of CH for 24 h. The sample was then dried in a 60 °C oven for 24 h.

### 2.7 Conductivity Measurements

A welded cylinder of EMImAc-laden Aida cloth was cut into ca. 1 in. × 1 in. pieces, and each piece was submerged in 100 mL of 18.2 MΩ·cm H_2_O. The vessel was placed on a rotary table to mix, and the solution was replaced with 100 mL of fresh H_2_O at various time intervals (the conditions for EMImAc extraction are discussed below). In each case, the conductivities of the rinse solutions were measured to monitor the extraction progress.

#### 2.7.1 Time Variance

**Scheme 1:**
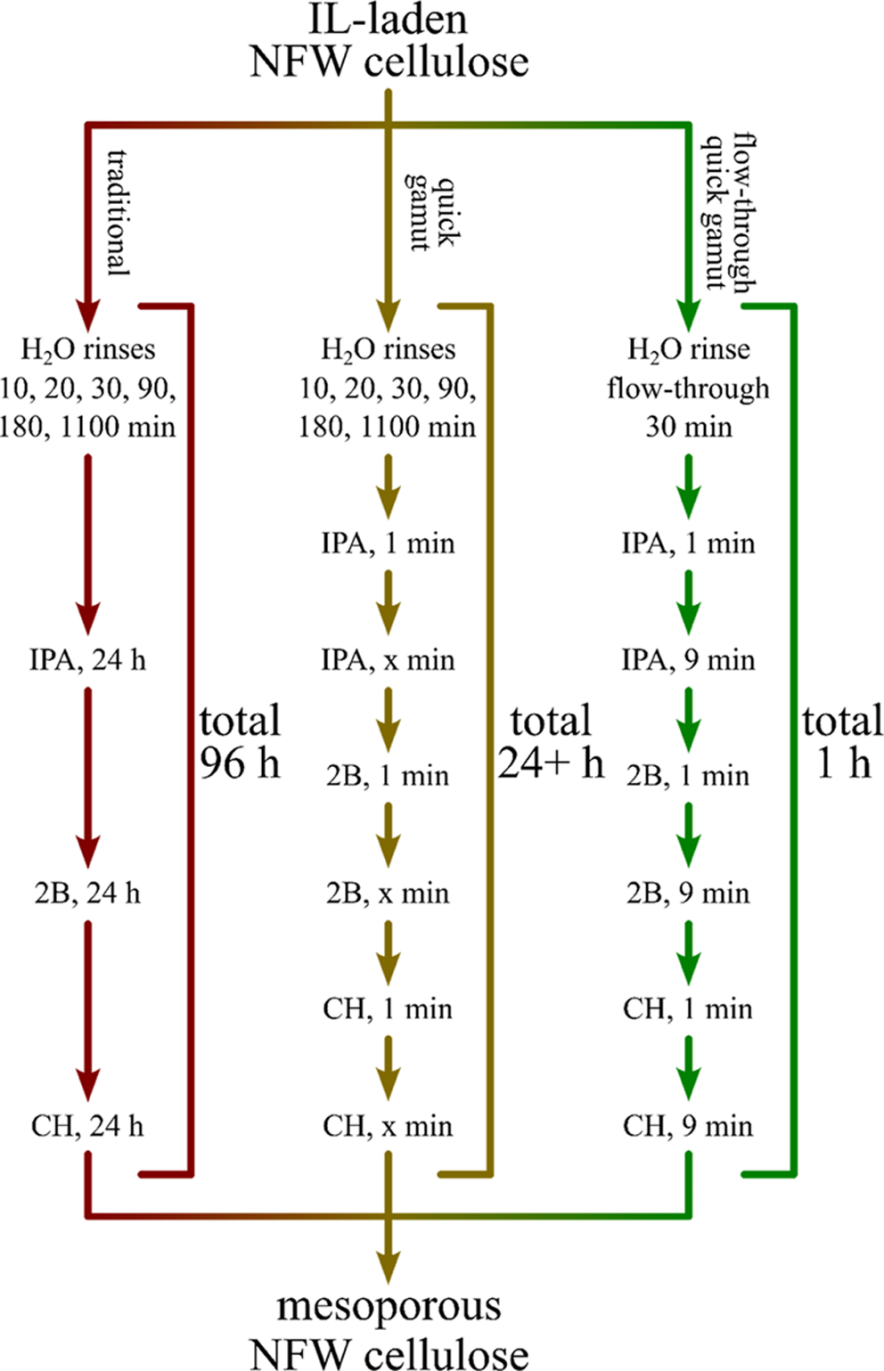
Flow Diagram Depicting the Three Rinse Techniques: Traditional (96 h Total), Quick Gamut (24–26 h), and Flow-Through with Quick Gamut (1 h) Note that *x* = 4, 9, 14, or 29.

Four trials were completed by using rinse intervals of 1, 5, 10, or 20 min (Scheme 1). In total, 10 rinse solutions were collected for each sample. The welded cloth could then reside in fresh H_2_O for a total exposure time of 24 h before being retrieved, subjected to the sequential gamut of solvents, dried in an oven at 60 °C, and analyzed by BET.

#### 2.7.2 Temperature Variance

Three trials were completed by performing 10 H_2_O rinses (5 min intervals) at 22, 45, or 60 °C. Note that a bulk of 18.2 MΩ·cm H_2_O was maintained at the required temperature, and 100 mL aliquots were retrieved for the rinses. Accordingly, the solvent replenishment for each 5 min rinse was at the desired temperature and was then allowed to cool naturally prior to being replaced. The conductivity of each 5 min rinse solution was recorded (measurements were performed with room temperature solutions), and the welded cloth was allowed to rest in fresh room temperature H_2_O for a total exposure time of 24 h before being retrieved, subjected to the sequential gamut of solvents, dried in an oven at 60 °C, and used for BET analysis.

### 2.8 Quick Gamut Treatment

An accelerated rinse process was developed to minimize the solvent exchange time. In short, H_2_O-rinsed welded cloth was submerged sequentially in IPA, 2B, and CH (i.e., the typical “gamut” of rinses) to achieve a mesoporous biopolymer composite. However, we replaced the traditional 24 h immersion with shorter rinse times, labeled herein as “1 + *x* min” for clarity (see Scheme 1). For example, a 1 + 4 min rinse was performed by first submerging the H_2_O-laden composite in IPA for 1 min, then the solvent was refreshed, and the composite was soaked for 4 additional min. This process was then repeated for 2B using the now IPA-laden composite and then CH using the 2B-laden composite. In total, trials for 1 + 4, 1 + 9, 1 + 14, and 1 + 29 min were performed. All samples were dried in an oven at 60 °C after the final rinse.

### 2.9 Flow-Through System

A flow-through system was fabricated using a Cole Parmer Masterflex L/S pump (Model 7554–90) with PTFE tubing and a 47 mm Continuous Flow Filtration Kit (167210–36) with a steel filter to hold the welded Aida cloth sample in place. A photograph of this setup is provided in Figure S1. Two trials were completed using this flow system with residence times of 20 or 30 min, collecting eluent, and measuring conductivity every 5 min. The samples were then put through a quick gamut rinse (see above) with immersion times of 1 + 9 min.

## 3 Results and Discussion

### 3.1 Conductivity as a Means of Measuring EMImAc Removal

**Figure 1:**
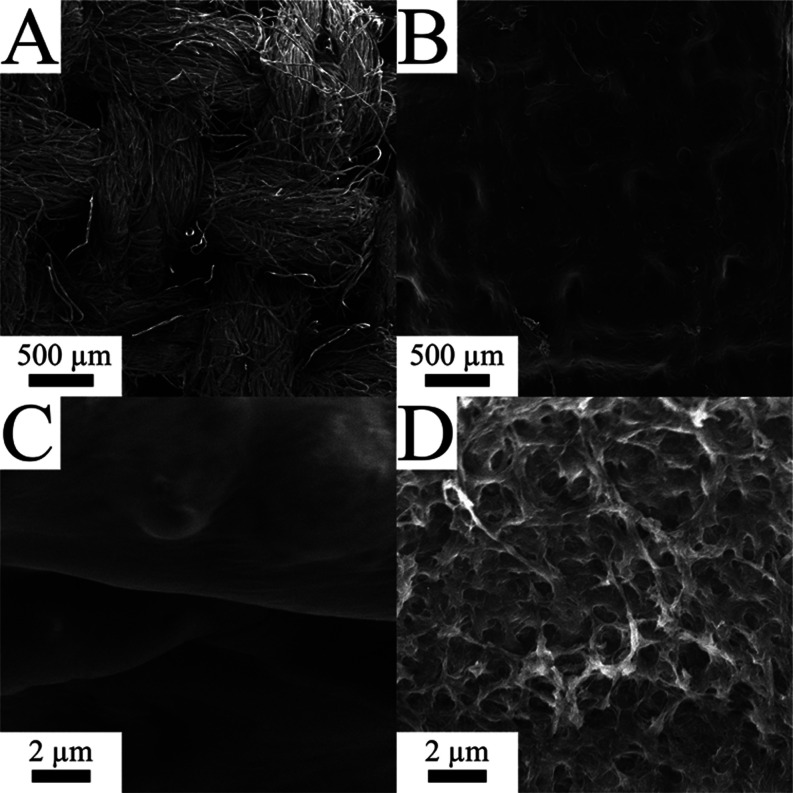
SEM images depicting (A, C) nonwelded Aida cloth and (B, D) mesoporous NFW Aida composites prepared using the traditional rinse method (24 total H_2_O rinses and 24 h per solvent during the gamut: IPA-2B-CH).

**Figure 2:**
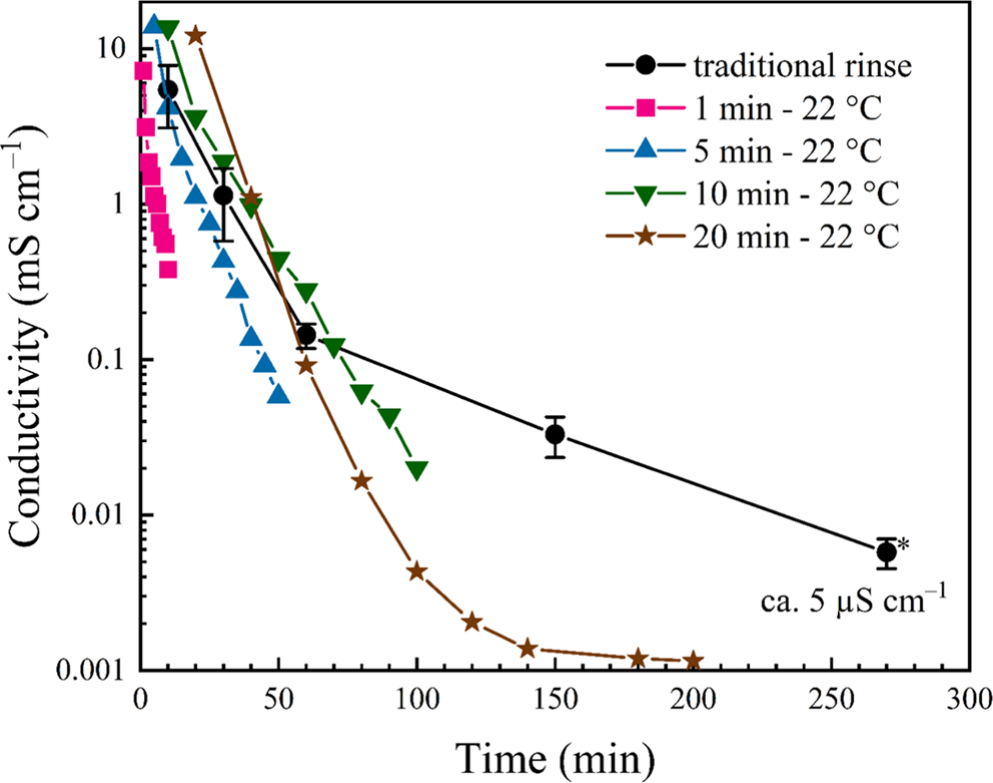
Plot comparing the conductivities of H_2_O rinse solutions from EMImAc-laden NFW Aida cloth when varying the soak duration. The black line represents the conductivities of rinse solutions during a traditional water rinse. Ten rinses were completed for the other four trials, and all rinses were performed using room temperature (ca. 22 °C) H_2_O.

The initial NFW mesoporous cotton studies utilized a single layer of abutting Coats and Clark cotton thread wrapped around a Teflon scaffold.^16,17^ This structure is noticeably thinner than the woven 22 count Aida cloth used herein (0.18 mm for the thread versus 0.26 mm thickness for the Aida cloth). Due to this difference, we started with a typical NFW experiment and gamut rinse to obtain a benchmark mesoporous Aida cloth sample. The resulting composite had a surface area of 163.2 m^2^ g^–1^ and an average pore diameter of 9.6 nm (Figure S4); these values are the benchmarks for each experiment to follow. SEM images of nonwelded Aida cloth depict loose woven fibers (Figure 1A) and a smooth texture (Figure 1C). Meanwhile, images of the traditional rinsed NFW mesoporous Aida cloth depict a welded mat (Figure 1B) with a mesoporous texture (Figure 1D). Additionally, the conductivity of each aqueous rinse solution was measured (Figure 2, labeled “traditional rinse” in black). The final rinse solution—assumed clean by traditional methods—exhibited a conductivity of ca. 6 μS cm^–1^; this will be the target “clean” value when measuring the conductivities of iterative or continuous flow H_2_O rinses/eluent.

EMImAc removal was interrogated by rinsing freshly welded Aida composites with H_2_O and performing 10 refreshments in 1, 5, 10, or 20 min intervals. Interestingly, iterative 20 min rinses resulted in a clean rinse after 100 min (i.e., 5 rinses; Figure 2), while shorter immersion times trend toward a faster decrease in conductivity. Indeed, the fastest rate of IL removal is achieved by the 1 min rinse schedule, though this is certainly the most solvent-intensive outcome, given that 10 rinses with 1 min intervals did not drop below a conductivity of 300 μS cm^–1^. From these data, we hypothesize that a flow-through (i.e., infinitely refreshing turnover) system is necessary to optimize EMImAc removal and alleviate the inefficiency of successive solvent rinses.

**Figure 3:**
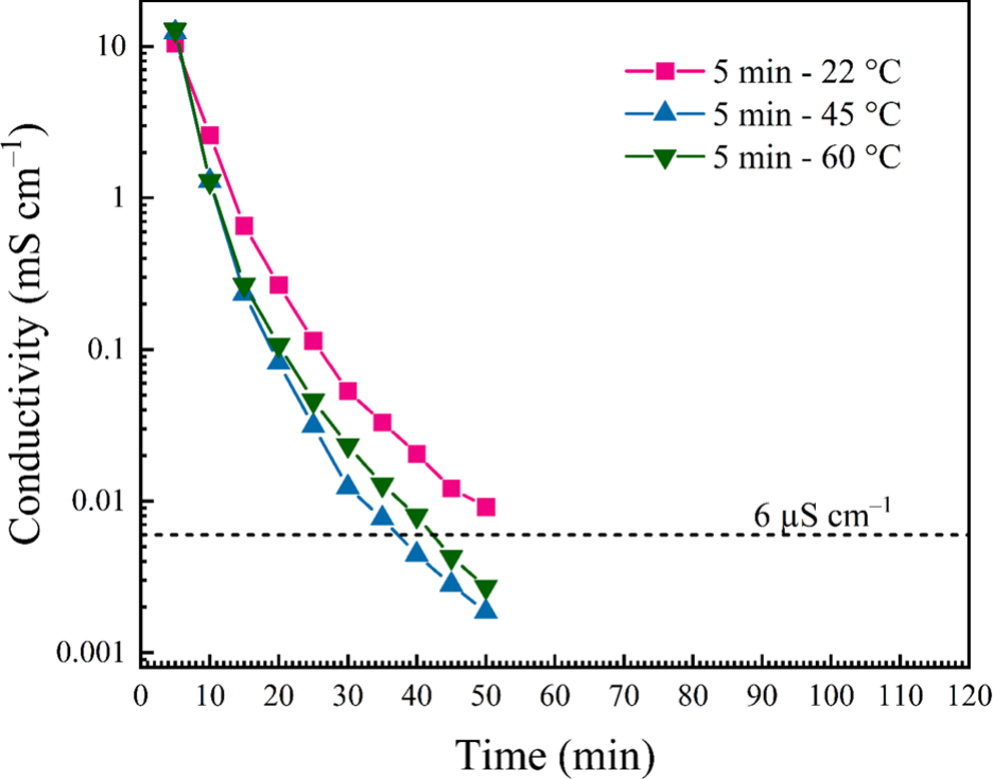
Plot comparing the conductivity of rinse solutions from EMImAc-laden NFW Aida cloth using warm H_2_O. In each case, a reservoir of H_2_O was maintained at the indicated temperature, and 200 mL aliquots were used to replenish the rinse solutions. Note that these aliquots were allowed to cool naturally toward ambient temperature upon addition of NFW Aida cloth (i.e., temperature maintenance was not performed on the extraction solutions once cloth was added).

Rinse solvent temperature is an important consideration for efficient solvent exchange. To interrogate this apparent variable, two 5 min rinse regiments were performed using 45 or 60 °C H_2_O. Succinctly, higher temperatures do increase extraction efficiency (i.e., faster decrease in conductivity rinse-over-rinse), with the 45 and 60 °C both yielding faster extraction rates compared to experiments performed at room temperature (Figure 3). However, BET analysis of these materials reveals that higher temperatures impart a detrimental effect on the surface area and pore size of a composite (Figure S4). For example, the 10 sequential 5 min 60 °C extractions result in a loss of ca. 85% of the BET surface area versus the comparable room temperature extraction. As the ultimate goal of this study is to optimize the production of mesoporous composites, room-temperature solvents were used for all remaining experiments.

### 3.2 Implementing the Quick Gamut Rinse Procedure

**Figure 4:**
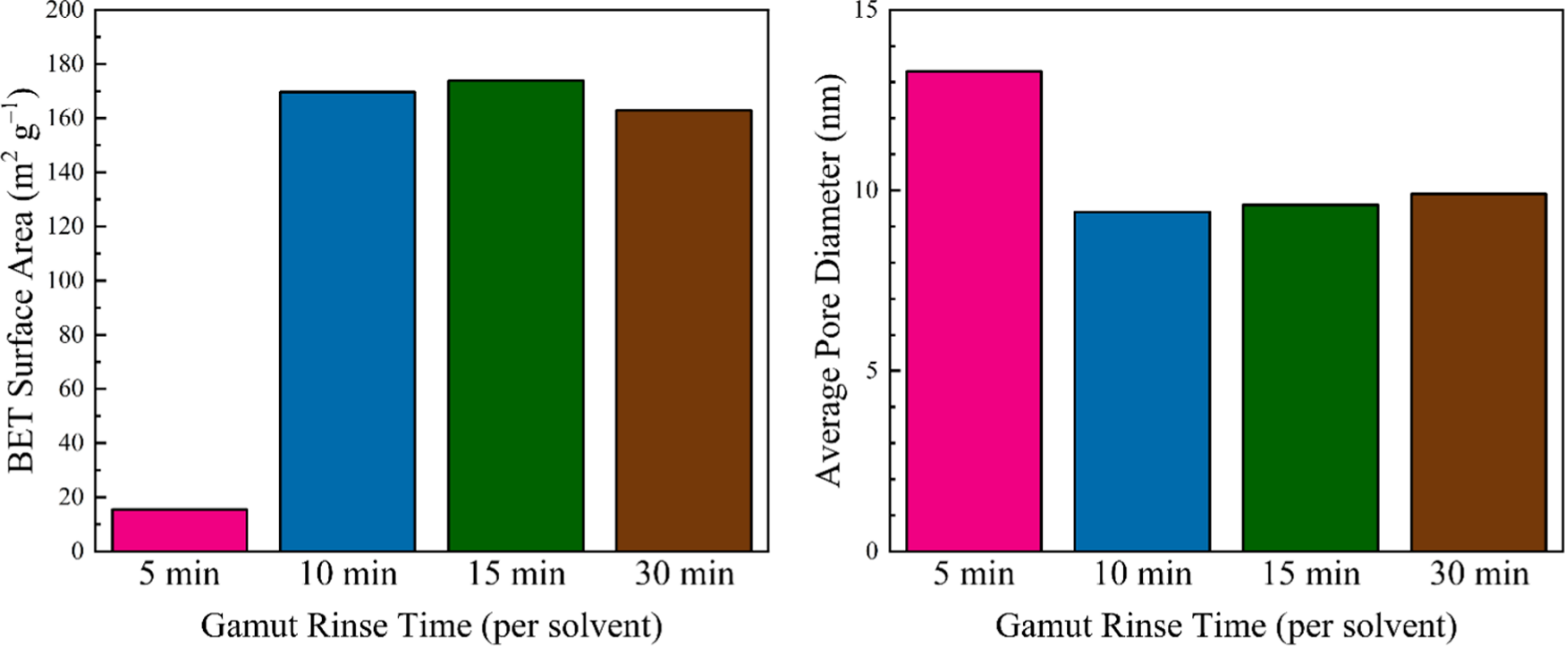
Plots illustrating the BET surface areas (left) and average pore diameters (right) when performing quick gamut rinses on traditional H_2_O-rinsed mesoporous cotton. Note that each time designation is the total time per solvent when using a 1 min refresh to remove most of the previous solvent (e.g., the 5 min rinse is 1 + 4 min, where solvent is refreshed after 1 min of exposure).

A traditional gamut series of rinses require 24 h per non-H_2_O solvent (namely, IPA, 2B, and CH), leading to a lengthy 72 h process. This was shortened by performing rinses of 1 + 4, 1 + 9, 1 + 14, and 1 + 29 min (1 min initial exposure followed by a second period of longer exposure; labeled as 5, 10, 15, and 30 min rinses) per solvent (Scheme 1 and Figures 4 and S5). Interestingly, the composite surface areas and pore dimensions are largely unaffected by solvent exposure times of ≥10 min. In addition, 5 min is likely too short (i.e., solvent exchange within the NFW Aida cloth occurs on a time scale larger than 5 min), as it results in a loss of ca. 90% of the surface area and ca. 40% larger pores. Overall, the shorter immersion time is excellent for process acceleration, and we can enhance this parameter further by using a flow-through system.

### 3.3 Flow-Through Mesoporous Cotton Production

**Figure 5:**
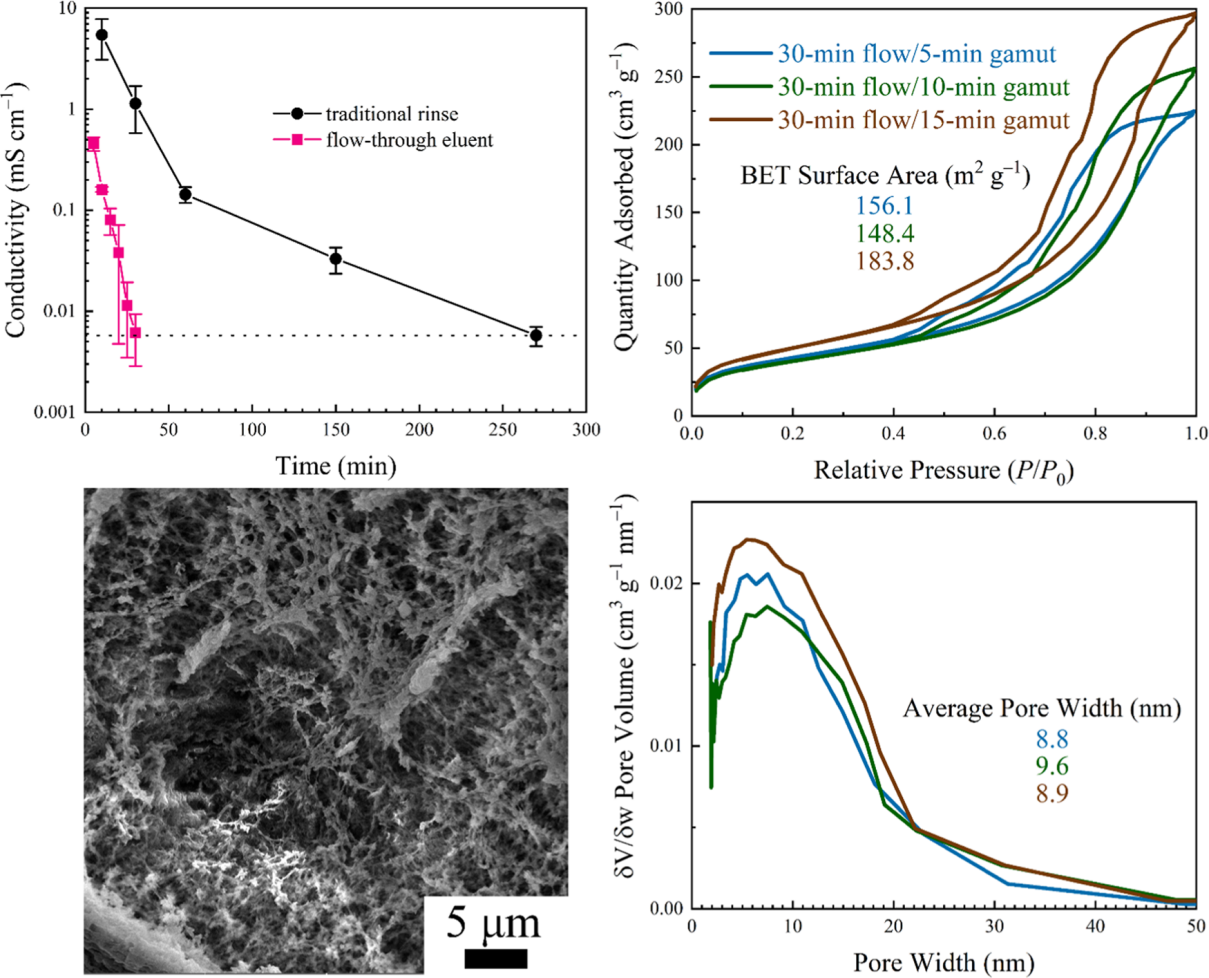
Panels depicting the results for the flow-through H_2_O rinse and quick gamut. (Top left) Conductivity measurements (and comparison to the traditional rinse) of *n* = 3 flow-through H_2_O rinses (flow rate of 0.6 mL min^–1^) where eluent was collected and measured every 5 min. The blue dashed line represents the target conductivity of 6 μS cm^–1^. (Top right) BET N_2_ sorption plot and (bottom right) pore size distributions for the flow-through and quick gamut analyses (both right panels use the same legend). (Bottom left) Example SEM image of the 30 min flow/15 min quick gamut sample to demonstrate the mesoporous structure.

A peristaltic pump flow-through system was assembled to draw H_2_O from a reservoir and continuously and controllably pass it through a 1 in. × 1 in. square of IL-laden NFW cloth. The impinging flow was maintained at a rate of 0.6 mL min^–1^, which was rapid enough to produce a small pool of solution in the sample reservoir while also maintaining a constant flow of waste eluent from the apparatus. Notably, this eluent could be easily collected for the IL recovery. Eluent conductivity was measured every 5 min, and the target eluent conductivity of ca. 6 μS cm^–1^ was achieved after 30 min of continuous flow (Figure 5, top left panel), corresponding to just 18 mL of H_2_O. To confirm the removal of absorbed IL (i.e., not just the coating of IL on the composite surface), a 30 min flow-rinsed composite was immersed in H_2_O for 24 h, achieving a solution conductivity of ca. 6.2 μS cm^–1^ and indicating that the flow-through system is adequate for rinsing IL. The 30 min flow-through rinse was then applied alongside 5, 10, and 15 min quick gamut rinses to create mesoporous Aida cloth composites. All composites give similar surface areas (ca. 150–180 m^2^ g^–1^; Figure 5, top right panel) and average pore size (ca. 9–10 nm; Figure 5, bottom right panel). The success of the 5 min gamut rinse is unexpected due to the poor performance of a similar sample during the quick gamut experiments, so it is likely that an amount of time between 5–10 min is optimal; it is recommended to move toward a longer exposure time to ensure an adequate solvent exchange. SEM imaging of the 30 min flow-through rinse, 10 min gamut composite (Figure 5, bottom left panel) reveals the expected highly mesoporous structure and is representative of all SEM images of the flow-through samples.

## Conclusions

Herein, we demonstrate the process acceleration of NFW toward the creation of mesoporous cellulose composites, reducing the time required by the traditional rinsing methodology by almost 99% (from ca. 96 to ca. 1 h). EMImAc removal from welded Aida cloth was monitored using the conductivity of aqueous rinse solutions, with a targeted final conductivity of ca. 6 μS cm^–1^. Using conductivity measurements, it was determined that a quick rinse solution turnover correlates to faster IL removal, reinforcing the need for continuous flow (i.e., infinite dilution). Next, a rapid solvent exchange (the quick gamut; IPA-2B-CH) was developed to minimize immersion time during solvent exchanges; based on BET surface areas and pore distributions, a 10 min gamut rinse is sufficient to achieve the BET surface area of ca. 150–180 m^2^ g^–1^ and average pore width of 9–10 nm typical of all-cellulose NFW composites. Finally, these modifications were combined with a peristaltic pump in a continuous flow system (flow rate of 0.6 mL min^–1^) for the aqueous rinses, achieving the target conductivity after 30 min using only ca. 18 mL total H_2_O. By finishing with a 10 min per solvent gamut, we achieve the target mesoporous NFW composite in just 1 h, a significant improvement over the 96 h (!) required by the traditional treatment.^17^ Adjustments to this assembly can allow for easy reclamation of wet EMImAc from the eluent stream, channel switching to other solvent reservoirs, or reactant input for automated derivatization of NFW cellulose (e.g., acetylation^25,26^ or TEMPO^27^). Overall, this contribution toward the rapid turnover of mesoporous all-cellulose NFW composites—materials with enticing nanomaterial encapsulation properties and the potential for separations/filtering, catalytic, and textile applications—extends their viability for researchers while simultaneously increasing the potential for commercialization of functional NFW.

## Supporting Information Available

The Electronic Supporting Information contains photographs of the sample and rinsing apparatus and BET adsorption hysteresis and pore distribution plots (PDF)
