# Supplementary material for: Accelerated Fabrication of Fiber-Welded Mesoporous Cotton Composites
Source: ACS Omega. 2024 Feb 9;9(7):8503–9. doi: 10.1021/acsomega.3c09797 (PMC10882613; doi:10.1021/acsomega.3c09797)
Supplement: Supplementary file 1 — ao3c09797_si_001.pdf [file ao3c09797_si_001.pdf]

Accelerated Fabrication of Fiber Welded Mesoporous Cotton Composites

Peyton J. Johnson[1], Anders J. Gulbrandson[1], Nathaniel E. Larm[1], Christopher D. Stachurski[1], David P. Durkin[1], and Paul C. Trulove[1,*]

[1] Department of Chemistry, United States Naval Academy, Annapolis, MD 21402, United States

*Email: trulove@usna.edu

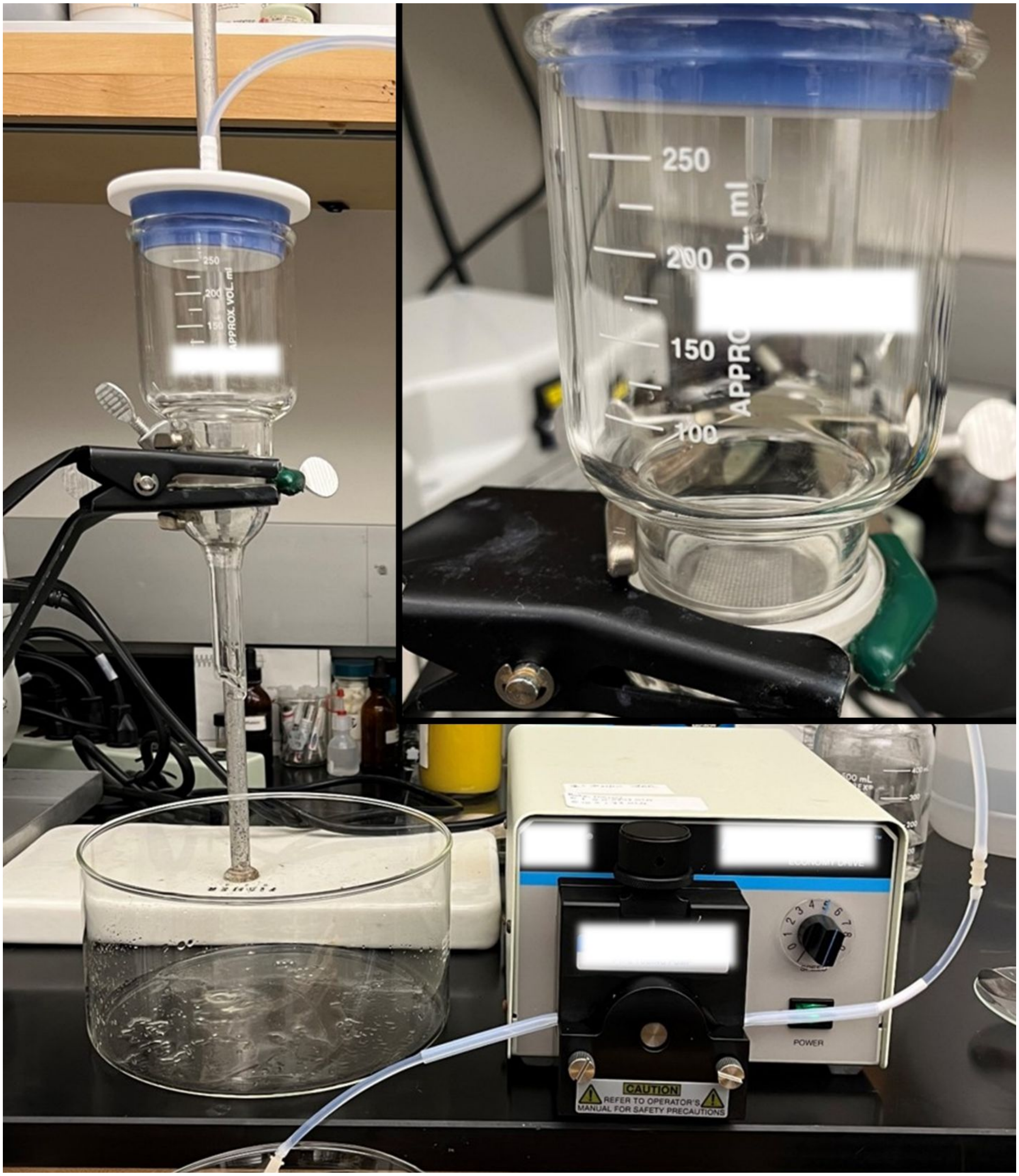

Figure S1. Photograph of the peristaltic pump H_2O flow-through rinse setup. 18.2 MΩ·cm H_2O is pumped from a reservoir (beaker) to a filtration setup with a piece of EMImAc-laden welded cotton held between a glass frit and stainless-steel grating (inset). Eluent is collected for analysis or discarded.

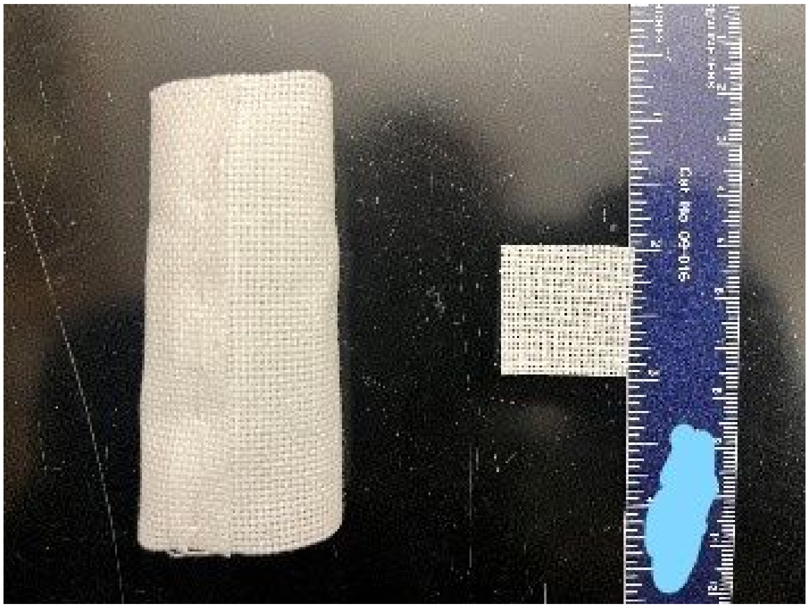

Figure S2. Photograph of (left) a cylindrical piece and (right) a 1” × 1” cutting of Aida cloth. The cylindrical piece was originally a cut measuring 3” × 4” which was then sewn into a cylinder with Coats cotton thread along the shorter edge.

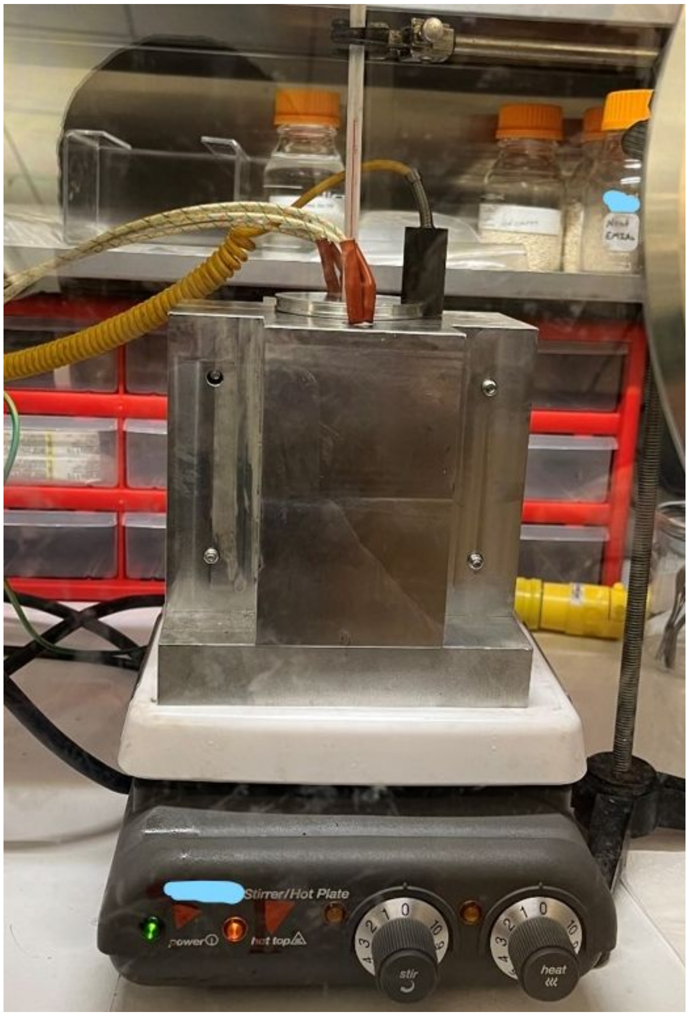

Figure S3. Photograph of the custom aluminum heating block enclosure. Within is a 240-mL Qorpak bottle containing the welding solution (ca. 200 mL of EMImAc).

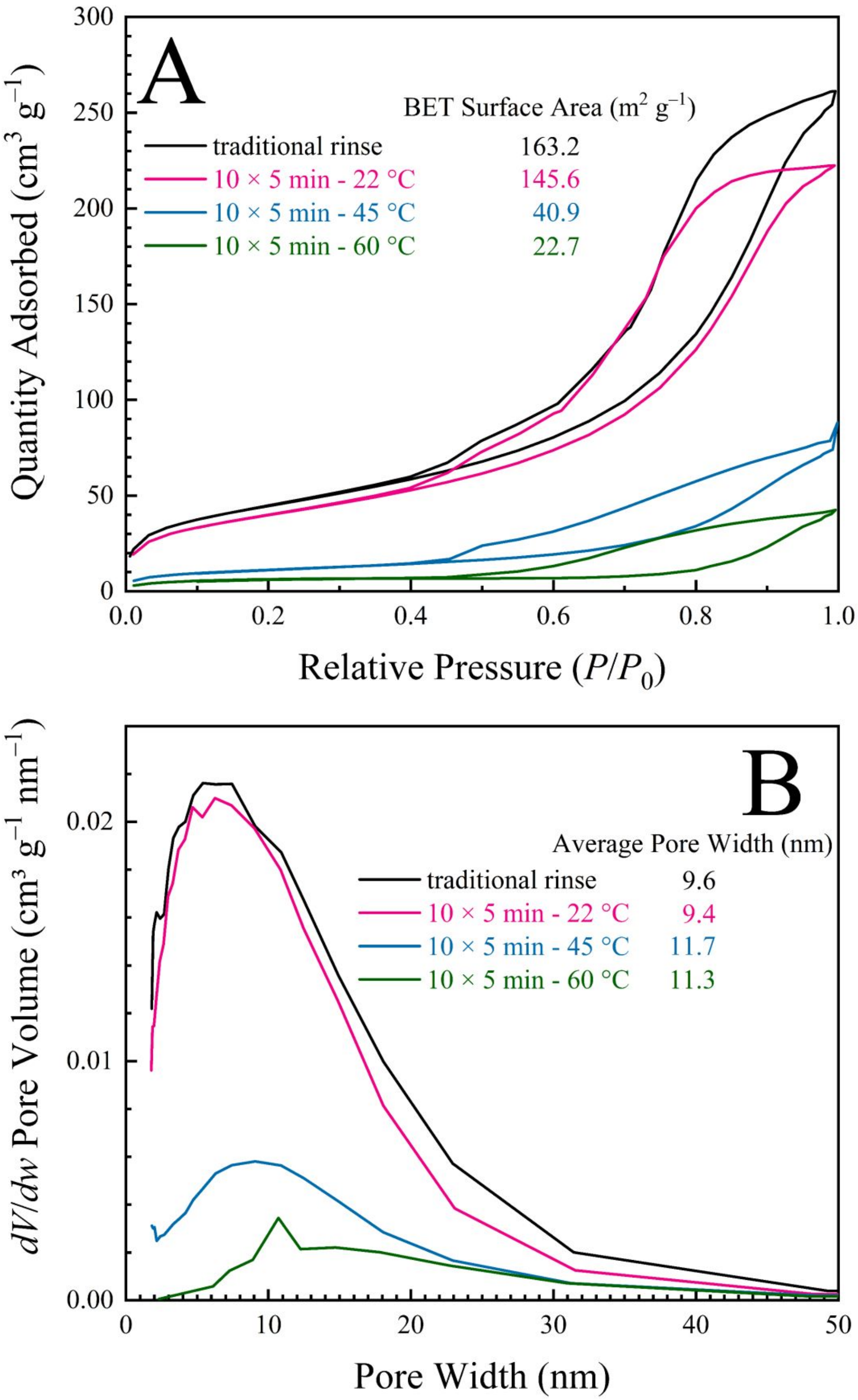

Figure S4. BET N_2 gas physisorption (A) hysteresis and (B) pore distribution plots for the temperature variance experiment using 10 rinses of 5 min each. Notably, temperatures greater than 22 °C result in faster EMImAc removal but also lower surface areas and larger pores when compared to a traditional rinse regiment.

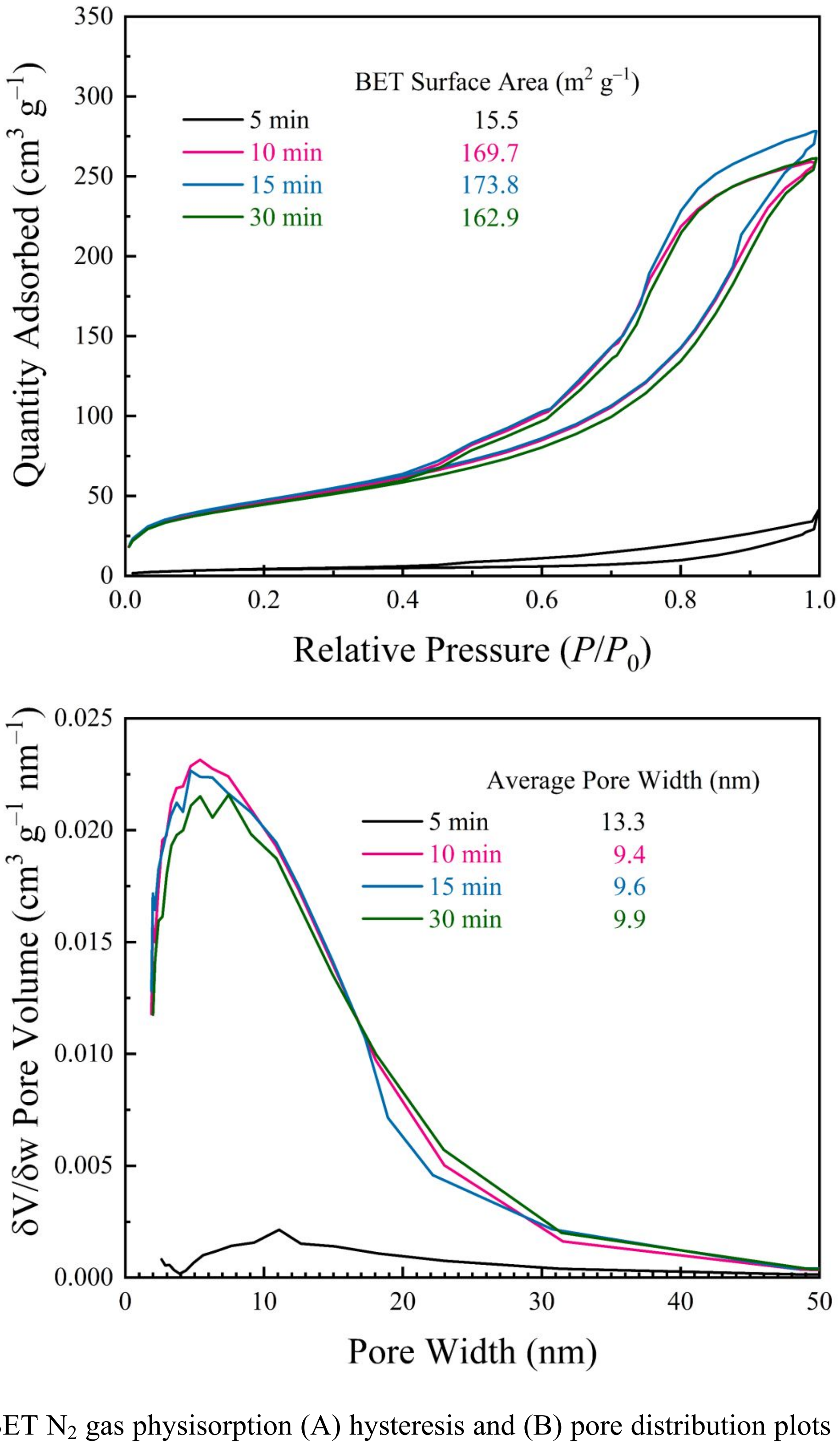

Figure S5. BET N_2 gas physisorption (A) hysteresis and (B) pore distribution plots for the quick gamut rinses. Note that the durations listed are actually the summation of exposure time (e.g., the 10 min sample was submerged in each solvent for 1+9 min, where the solvent was refreshed after 1 min to remove most of the residual solvent from the previous exposure).
